# Hardening of a dual-cure resin cement using QTH and LED curing units

**DOI:** 10.1590/S1678-77572010000200003

**Published:** 2010

**Authors:** Maria Jacinta Moraes Coelho SANTOS, Sheila Pestana PASSOS, Monalisa Olga Lessa da ENCARNAÇÃO, Gildo Coelho SANTOS, Marco Antonio BOTTINO

**Affiliations:** 1 DDS, MSc, PhD, Associate Professor, Department of Restorative Dentistry, University Western Ontario.; 2 DDS, MSc, Graduate student, Department of Dental Materials and Prosthodontics, São José dos Campos Dental School, São Paulo State University, São José dos Campos, SP, Brazil.; 3 DDS, Department of Dental Materials and Prosthodontics, Dental School, Federal University of Bahia, Salvador, BA, Brazil.; 4 DDS, MSc, PhD, Associate Professor, Department of Dental Materials and Prosthodontics, São José dos Campos Dental School, São Paulo State University, São José dos Campos, SP, Brazil.

**Keywords:** Hardness, Cure, Resin cements

## Abstract

**Objective:**

This study evaluated the surface hardness of a resin cement (RelyX ARC) photoactivated through indirect composite resin (Cristobal) disks of different thicknesses using either a light-emitting diode (LED) or quartz tungsten halogen (QTH) light source.

**Material and Methods:**

Eighteen resin cement specimens were prepared and divided into 6 groups according to the type of curing unit and the thickness of resin disks interposed between the cement surface and light source. Three indentations (50 g for 15 s) were performed on the top and bottom surface of each specimen and a mean Vickers hardness number (VHN) was calculated for each specimen. The data were analyzed using two-way ANOVA and Tukey-Kramer test was used for post-hoc pairwise comparisons.

**Results:**

Increased indirect resin disk thickness resulted in decreased mean VHN values. Mean VHN values for the top surfaces of the resin cement specimens ranged from 23.2 to 46.1 (QTH) and 32.3 to 41.7 (LED). The LED curing light source produced higher hardness values compared to the QTH light source for 2- and 3-mm-thick indirect resin disks. The differences were clinically, but not statistically significant. Increased indirect resin disk thickness also resulted in decreased mean VHN values for the bottom surfaces of the resin cement: 5.8 to 19.1 (QTH) and 7.5 to 32.0 (LED). For the bottom surfaces, a statistically significant interaction was also found between the type of curing light source and the indirect resin disk thickness.

**Conclusions:**

Mean surface hardness values of resin cement specimens decreased with the increase of indirect resin disk thickness. The LED curing light source generally produced higher surface hardness values.

## INTRODUCTION

Due to their excellent esthetic and superior mechanical properties, resin cements are considered the material of choice to be used with metal-free restorations^10^. The mechanical properties and biocompatibility of resin cements are directly related to the degree of monomer conversion^16^. Several studies have demonstrated that the degree of monomer conversion determines the surface hardness and wear resistance of the resin materials^16,20^. Maximum monomer conversion is always desired to ensure optimum properties and biocompatibility and to reduce water solubility^7,37^. However, total monomer conversion with resin polymers is virtually unattainable and these materials always display some residual monomer after polymerization.

Dual cured resin cements have been advocated for luting ceramic restorations because they do not adversely affect esthetics and they allow adequate working time to complete the procedure. However, the amount or degree of conversion of the resin cement may vary, especially with bulky restorations thereby compromising the retention of the crown or inlay restoration^9,11,12,15,20,31^. If a photo-cured or dual cured resin material does not receive a sufficient number of photons at the correct wavelength, the amount of polymerization and degree of conversion will be inadequate^25^. Furthermore, other studies have reported an inverse relationship between the thickness of ceramic inlays and the surface hardness of light-cured and dual cured resin cements^12,15^.

The polymerization process of composite materials can be accomplished with different light sources. Quartz tungsten halogen (QTH) curing units are currently the most commonly used means of curing dental composites. However, this technology has several drawbacks, such as a limited lifespan (40-100 h) and the generation of high temperatures during light emission. This results in a degradation of the bulb, reflector and filter over time and reduction of the QTH curing effectiveness^36^. To overcome these problems, new light-sources have been developed and introduced to the market, such as, plasma arc (PAC), laser lights and light-emitting diode (LED)^26,37^.

Although the first generation of LED curing lights resulted in insufficient polymerization of composite resins^8,24,33^, newer versions of LED units deliver a spectral emission with greater peak irradiance and power output. Some studies have shown that LED is now as effective as QTH curing light units^4,17,28,30^. LED units have an expected lifetime of several thousand h without significant degradation of light flux over time and no filters are required, since their spectral output falls conveniently within the absorption spectrum of the camphoroquinone photoinitiator (400-500 nm)^18,31,34^.

The aim of this investigation was to evaluate the surface hardness of a dual-cure resin cement (RelyX ARC) cured using QTH and LED curing light sources through indirect resin disks of different thicknesses. The null hypotheses tested were: 1- There is no difference in the surface hardness of the resin cement cured through indirect resin disks of different thickness; 2- There is no difference in the surface hardness of resin cement cured with a LED light source compared to a QTH light source.

## MATERIAL AND METHODS

Disks measuring 5 mm in diameter and thicknesses of 1, 2 and 3 mm were fabricated with an indirect composite resin (Cristobal; Microdont São Paulo, SP, Brazil) according to the manufacturer’s instructions. Then, eighteen 2mm-thick specimens were prepared from a dualcure resin cement (RelyX ARC; 3M/ESPE, St. Paul, MN, USA) according to the manufacturers’ instructions for ratio and mixing. For each specimen, a ring placed on a glass slide lined with a mylar polyester strip was filled with the resin cement and covered with another mylar strip. Then, an indirect composite resin disk (1, 2 and 3 mm thick) was placed onto this set, and the resin cement was photoactivated through the resin disk for 40 s with one of the two curing light sources: LED (Elipar^TM^ FreeLight 2 LED Curing Light; 3M/ESPE; 800 mW/cm^2^) or QTH (Optilight Plus; Gnatus, Ribeirão Preto, SP, Brazil; 500 mW/cm^2^). Three specimens were prepared for each test condition, forming 6 groups: 1- QTH + 1-mm-thick indirect resin disk; 2- LED + 1mm-thick indirect resin disk; 3- QTH + 2-mmthick indirect resin disk; 4- LED + 2-mm-thick indirect resin disk; 5- QTH + 3-mm-thick indirect resin disk; 6- LED + 3-mm-thick indirect resin disk. All specimens were stored dry in boxes in a darkened incubator at 37°C for 24 h before testing.

A hardness test using a Vickers diamond indenter (Digital Hardness Tester FM, Future-Tech, Tokyo, Japan) was performed on the surface of each specimen with a 50-g load for 15 s. Three indentations were obtained for each of the upper and the lower surfaces of each resin cement specimen. Mean Vickers hardness numbers (VHN) were then calculated for both surfaces. The data were analyzed by two-way ANOVA. If a statistically significant difference was observed among the groups, a Tukey- Kramer test was used to determine pair-wise differences. A pvalue of 0.05 or less was considered statistically significant, and a difference in mean VHN hardness values of 20% or greater was considered to be clinically meaningful.

## RESULTS

Mean Vickers hardness values (VHN) and standard deviations for the top surface of the resin cement specimens are presented in Table 1. For both types of curing light sources, the mean VHN values for the top surface of the resin cement specimens decreased as the indirect resin disk thickness increased. The VHN of the top surface of the resin cement was 22.5% lower when a 3mm-thick indirect resin disk was used compared to when a 1-mm-thick indirect resin disk was used for the LED unit, and 49.7% lower for the QTH light source. The QTH light source produced a slightly greater VHN value (12.1%) compared to the LED light source using the 1-mm-thick indirect resin disk, but lower VHN values with the 2- and 3-mm-thick indirect resin disks (36.3% and -47.0%, respectively). The differences in surface hardness values for the 2and 3-mm-thick indirect resin disk were clinically meaningful in favor of the LED curing light source. There was no interaction between the type of curing light source and the indirect resin disk thickness for the top surface of the resin cement specimens. No statistically significant difference was found in the mean VHN values for the top surface of the resin cement (p= 0.24) between the two types of curing light sources. There was, however, a statistically significant difference in mean hardness among the indirect resin disk thicknesses (p=0.01). A Tukey-Kramer test revealed that this difference was statistically significant (p< 0.05) between the 1- and 3-mmthick indirect resin disks, but not between the 1and 2-mm-thick disks or between the 2- and 3mm-thick disks.

**Table 1:** Mean hardness values (VHN) (standard deviation) for the top surface of the resin cement specimens varying the curing light source and thickness of the indirect resin disks

**Resin disk thickness (mm)**	**Light Source**	Top *	**% Difference Compared to 1 mm**	% Difference ** by curing light source and thickness
1.0	QTH	46.1 (9.89)ª	-	12.1%
1.0	LED	41.7 (1.95)^ab^	-	
2.0	QTH	27.0 (11.98)^ab^	-41.4%	36.3%
2.0	LED	36.8 (2.80)^ab^	-11.8%	
3.0	QTH	23.2 (11.27)^b^	-49.7%	47.0%
3.0	LED	32.3 (2.31 )^ab^	-22.5%	

* Same superscripted letters indicate no statistically significant differences (p > 0.05).

** Absolute difference in mean VHN/ mean QTH VHN.

Mean VHN means and standard deviations for the bottom surface of the resin cement specimens are presented in Table 2. The LED light source produced slightly greater VHN values compared to the QTH light source when used with all three indirect resin disk thicknesses. The VHN values on the bottom surface decreased dramatically with the increase of the indirect resin disk thickness for both types of curing light sources. The mean VHN value on the bottom surface of the resin cement was 76.6% lower when a 3mm-thick indirect resin disk was used compared to a 1-mm-thick indirect resin disk was used for the LED light source and 69.6% lower for the QTH light source. There was statistically significant difference between the mean VHN values for the type of curing light source (p= 0.03) and, similarly, for the indirect resin disk thicknesses (p< 0.001). However, there was also a statistically significant interaction (p= 0.009) when the type of curing light source and the indirect resin disk thickness were combined. The LED light source using a 1-mm-thick indirect resin disk produced the highest mean VHN value on the bottom surface, and the QTH light source using a 3-mm-thick indirect resin disk produced the lowest VHN value. The Tukey-Kramer test revealed that the difference in the mean VHN values for these two specific combinations was statistically significant (p< 0.05). The bottomto-top surface hardness ratios are shown in Table 3.

**Table 2:** Mean hardness values (VHN) (standard deviation) for the bottom surface of the resin cement specimens varying the curing light source and thickness of the indirect resin disks

**Resin diskt hickness (mm)**	**Light Source**	Bottom*	**% Difference Compared to 1 mm**	% Difference** by curing light source and thickness
1.0	QTH	19.1 (4.54)^b^	-	67.5%
1.0	LED	32.0 (3.28)ª	-	
2.0	QTH	11.4 (4.67)^b^	-40.3%	0.87%
2.0	LED	11.5 (1.84)^b^	-64.1%	
3.0	QTH	5.8 (1.60)^b^	-69.6%	29.3%
3.0	LED	7.5 (0.88)^b^	-76.6%	

* The same superscripted letters indicate no significant differences (p > 0.05).

** Absolute difference in mean VHN/ mean QTH VHN.

**Table 3:** Bottom-to-top surface microhardness ratio (%)

**Resin disk thickness (mm)**	**QTH**	**LED**
1.0	42.13	76.07
2.0	45.25	32.39
3.0	38.01	23.45

Generally, higher mean VHN values were obtained when the resin cement specimens were photoactivated with the LED curing light source. The mean VHS value decreased with the increase of the indirect resin disk thickness. The top surfaces of the resin cement specimens had consistently higher VHS values than the bottom surfaces.

## DISCUSSION

The surface hardness of cured resin materials can be a useful indicator of the degree of monomer conversion^2,21,30,32^. Uhl, et al.^34^ (2003) showed that the degree of polymerization of composite materials can be better evaluated with Knoop or Vickers hardness than with depth of cure tests using a penetrometer. Hardness tests may be classified based on the magnitude of indentation loads such as macrohardness, microhardness and nanohardness, being microhardness tests (Knoop, Vickers) the most common test used for composite materials^1^.

Adequate polymerization of resin cements materials may be a problem under indirect restorations^12^. According to Hasegawa, et al.^15^ (1991), the final hardness of the dual cured cements depends on the amount of exposure to the curing light. None of the dual cured resin cements tested in their study achieved complete hardening when not exposed to light, resulting in lower hardness as the chemically cured component did not provide complete hardening. This confirms the importance of light exposure to increase the hardness of dual cured cements.

The results of this study reject the first null hypothesis. Increased indirect resin disk thickness resulted in lower VHN values on both top and bottom surfaces of the resin cement specimens. These findings are similar to those of previous studies^9,12,19,23^. The maximum indirect resin disk thickness tested in this study was 3 mm, simulating the mean thickness of indirect restorations (2.5 mm). However, other studies have shown light obstruction beyond 4 mm thickness of indirect resin disks^12,23^.

Another important consideration related to the degree of resin polymerization is the light intensity delivered by the curing unit. Resin-based materials may present incomplete polymerization rate when light curing units with low outputs are used^8^. The ISO-recommended intensity for polymerization lights is 300 mW/cm^2^ ^16^. Light intensity of LED light curing units is fundamental for their good functioning^3^. In this study, light intensities of 500 and 800 mW/cm^2^ were delivered by the QTH and LED units, respectively.

The second null hypothesis of this study was also rejected. Higher VHN values were produced with the LED curing light source compared to the QTH curing light source, except for the one mm thick indirect resin disk. The sample size used in this study was insufficient to determine that the difference between the hardness values for the two light curing sources on the top surface of the resin cement with a one mm indirect resin disk was statistically significant. Cefaly, et al.^6^ (2009) evaluated the microhardness of RMGICs using LED and QTH units, and observed that when a LED light was used the microhardness values varied depending on the restorative material tested, in the same way as observed by Cefaly, et al.^5^ (2005) for resin-based materials. Franco, et al.^13^ (2007), Price, et al.^29^ (2003) and Dunn and Bush^8^ (2002) considered that QTH was superior to LED units for curing composites. For Kurachi, et al.^22^ (2001), the first generation of LEDs (6 diodes - 79 mW/cm^2^) reached 60% of the hardness achieved with QTH units (475 mW/ cm^2^), and reported that specimens cured with LED needed more exposure time to obtain the same depth of cure obtained with halogen light. This study used a high-power energy LED source. The first generation of LEDs presented approximately half of the power of the new generation^26^. Some authors^2,35^ have reported that high-power LED units present the same efficiency of QTH units, with the advantage of preventing overheating.

Uhl, et al.^35^ (2004), Uhl, et al.^36^ (2005) reported similar hardness values from composite material photoactivated with QTH and with second-generation LEDs (901 mW/cm^2^). These findings agree with those of Piva, et al.^28^ (2008), who used QTH (589 mW/cm^2^) and LED (614 mW/ cm^2^) units, and Gomes, et al.^14^ (2006). In the present study the LED curing light source generally produced higher surface hardness values, probably due to the higher energy density used in this group (LED - 24 J/cm^2^ versus QTH 15 J/cm^2^) and light intensity (QTH - 800 mW/ cm^2^ versus 500 mW/cm^2^) ^3^.

There is no internationally recognized standard for adequate depth of cure as measured by the relative hardness method^17^. For proper depth of cure, a relative hardness value (100 x hardness of lower surface/hardness of top surface) must be higher than 80%^27^. In this study, the bottomto-top surface hardness ratios were below 80% in all groups. This result is not in accordance with the study of Hooshmand, et al.^17^ (2009), who used 1-mm-thick specimens.

## CONCLUSIONS

Within the limitations of an *in vitro* investigation, the following conclusions can be reached: 1. For both the top and bottom surfaces of the resin cement specimens, the mean VHN values decreased as the thickness of the indirect resin disk thickness increased from 1 to 3 mm, irrespectively of the curing light source used; 2. Higher mean VHN values were found on the top surface compared to the bottom surface of the resin cement specimens regardless of the thickness of the indirect resin disk or the type of curing light source; 3. Except for the 1-mm-thick indirect resin disk, higher VHN values were produced with the LED unit compared to the QTH unit; 4. On the top surface of the resin cement specimens, the LED curing source produced significantly higher mean VHN values than the QTH light source when the cement specimens were photoactivated through 2- and 3-mm-thick indirect resin disks; 5. On the bottom surface of the resin cement specimens, there was a statistically significant interaction between the type of curing light source and the thickness of the indirect resin disks, that is LED curing source produced significantly higher mean VHN values than the QTH light source when the cement specimens were photoactivated through indirect resin disks thicker than 1 mm.

## References

[1] (2000). Mechanical testing and evaluation. ASM Handbook.

[2] Barghi N, McAlister EH (2003). LED and halogen lights: effect of ceramic thickness and shade on
curing luting resin. Compend Contin Educ Dent.

[3] Briso AL, Fedel TM, Pereira SM, Mauro SJ, Sundfeld RH, Sundefeld ML (2006). Influence of light curing source on microhardness of composite resins
of different shades. J Appl Oral Sci.

[4] Ceballos L, Fuentes MV, Tafalla H, Martínez A, Flores J, Rodríguez J (2009). Curing effectiveness of resin composites at different exposure times
using LED and halogen units. Med Oral Patol Oral Cir Bucal.

[5] Cefaly DF, Ferrarezi GA, Tapety CM, Lauris JR, Navarro MF (2005). Microhardness of resin-based materials polymerized with LED and
halogen curing units. Braz Dent J.

[6] Cefaly DF, Mello LL, Wang L, Lauris JR, D'Alpino PH (2009). Effect of light curing unit on resin-modified glass-ionomer cements: a
microhardness assessment. J Appl Oral Sci.

[7] Davidson-Kaban SS, Davidson CL, Feilzer AJ, Gee AJ, Erdilek N (1997). The effect of curing light variations on bulk curing and wall-to-wall
quality of two types and various shades of resin composites. Dent Mater.

[8] Dunn WJ, Bush AC (2002). A comparison of polymerization by lightemitting diode and
halogen-based light-curing units. J Am Dent Assoc.

[9] El-Badrawy WA, El Mowafy OM (1995). Chemical versus dual curing of resin inlay cements. J Prosthet Dent.

[10] El-Mowafy OM, Benmergui C (1994). Radiopacity of resin based inlay luting cements. Oper Dent.

[11] El-Mowafy OM, Rubo MH (2000). Influence of composite inlay/onlay thickness on hardening of
dual-cured resin cements. J Can Dent Assoc.

[12] El-Mowafy OM, Rubo MH, El-Badrawy WA (1999). Hardening of new resin cements cured through a ceramic
inlay. Oper Dent.

[13] Franco EB, Santos PA, Mondelli RF (2007). The effect of different lightcuring units on tensile strength and
microhardness of a composite resin. J Appl Oral Sci.

[14] Gomes GM, Calixto AL, Santos FA, Gomes OM, D'Alpino PH, Gomes JC (2006). Hardness of a bleaching-shade resin composite polymerized with
different light-curing sources. Braz Oral Res.

[15] Hasegawa EA, Boyer DB, Chan DC (1991). Hardening of dual cured cements under composite resin
inlays. J Prosthet Dent.

[16] Hofmann N, Papsthart G, Hugo B, Klaiber B (2001). Comparison of photo-activation versus chemical or dual curing of
resin-based luting cements regarding flexural strength, modulus and surface
hardness. J Oral Rehabil.

[17] Hooshmand T, Mahmoodi N, Keshvad A (2009). Microhardness of a resin cement polymerized by light-emitting diode
and halogen lights through ceramic. J Prosthodont.

[18] Jandt KD, Mills RW, Blackwell GB, Ashworth SH (2000). Depth of cure and compressive strength of dental composites cured with
blue light emitting diodes (LEDs). Dent Mater.

[19] Jung H, Friedl KH, Hiller KA, Furch H, Bernhart S, Schmalz G (2006). Polymerization efficiency of different photocuring units through
ceramic disks. Oper Dent.

[20] Jung H, Friedl KH, Hiller KA, Haller A, Schmalz G (2001). Curing efficiency of different polymerization methods, through ceramic
restorations. Clin Oral Inv.

[21] Koch A, Kroeger M, Hartung M, Manetsberger I, Hiller KA, Schmalz G (2007). Influence of ceramic translucency on curing efficacy of different
light-curing units. J Adhes Dent.

[22] Kurachi C, Tuboy AM, Magalhaes DV, Bagnato VS (2001). Hardness evaluation of a dental composite polymerized with
experimental LED-based devices. Dent Mater.

[23] Meng X, Yoshida K, Atsuta M (2006). Hardness development of dualcured resin cements through different
thickness of ceramics. Dent Mater.

[24] Mills RW, Uhl A, Jandt KD (2002). Optical power outputs, spectra and dental composite depths of cure,
obtained with blue light emitting diode (LED) and halogen light curing units
(LCUs). Br Dent J.

[25] Nomoto R (1997). Effect of light wavelength on polymerization of lightcured
resins. Dent Mater J.

[26] Nicoleta I, Kathrin F, Katja T, Reinhard H, Karl-Heinz K (2005). Shrinkage behavior of a resin-based composite irradiated with modern
curing units. Dent Mater.

[27] Pilo R, Cardash SH (1992). Post-irradiation polymerization of different anterior and posterior
visible light-activated resin composites. Dent Mater.

[28] Piva E, Correr-Sobrinho L, Sinhoreti MA, Consani S, Demarco FF, Powers JM (2008). Influence of energy density of different light sources on Knoop
hardness of a dual-cured resin cement. J Appl Oral Sci.

[29] Price RBT, Felix CA, Andreou P (2003). Evaluation of a Second-Generation LED curing light. J Can Dent Assoc.

[30] Rode KM, Kawano Y, Turbino ML (2007). Evaluation of curing light distance on resin composite microhardness
and polymerization. Oper Dent.

[31] Santos GC, El-Mowafy O, Rubo JH, Santos MJ (2004). Hardening of Dual-Cure Resin Cements Cured with QTH and LED Curing
Units. J Can Dent Assoc.

[32] Soares CJ, Silva NR, Fonseca RB (2006). Influence of the feldspathic ceramic thickness and shade on the
microhardness of dual resin cement. Oper Dent.

[33] Uhl A, Mills RW, Vowles RW, Jandt KD (2002). Knoop hardness depth profiles and compressive strength of selected
dental composites polymerized with halogen and LED light curing
technologies. J Biomed Mater Res.

[34] Uhl A, Mills RW, Jandt KD (2003). Photoinitiator dependent composite depth of cure and Knoop hardness
with halogen and LED light curing units. Biomaterials.

[35] Uhl A, Sigusch BW, Jandt K (2004). Second generation LEDs for the polymerization of oral
biomaterials. Dent Mater.

[36] Uhl A, Mills RW, Rzanny AE, Jandt KD (2005). Time dependence of composite shrinkage using halogen and LED light
curing. Dent Mater.

[37] Yoon TH, Lee YK, Lim BS, Kim CW (2002). Degree of polymerization of resin composites by different light
sources. J Oral Rehabil.

